# Red Meat Consumption and Cancer Risk: A Systematic Analysis of Global Data

**DOI:** 10.3390/foods12224164

**Published:** 2023-11-17

**Authors:** Hongyue Ma, Xiangming Qi

**Affiliations:** 1 State Key Laboratory of Marine Food Processing and Safety Control, College of Food Science and Engineering, Ocean University of China, Qingdao 266404, China; mahongyue@stu.ouc.edu.cn; 2 Haide College, Ocean University of China, Qingdao 266404, China; 3 Laboratory for Marine Drugs and Bioproducts of Qingdao National Laboratory for Marine Science and Technology, Qingdao 266237, China; 4 Qingdao Key Laboratory of Food Biotechnology, College of Food Science and Engineering, Ocean University of China, Qingdao 266404, China; 5 Key Laboratory of Biological Processing of Aquatic Products, China National Light Industry, Qingdao 266404, China

**Keywords:** red meat consumption, global data, cancer incidence, cancer risk, white meat consumption

## Abstract

The association between red meat consumption and cancer risk remains a controversy. In this study, we systematically collected and analyzed global data (from Our World in Data and Global Cancer Observatory) to investigate this association for the first time. Our results confirmed significant positive associations between red meat consumption (RMC) and overall cancer incidence (0.798, *p* < 0.001), or colorectal cancer incidence (0.625, *p* < 0.001). Several previously unreported cancer types linked to RMC were also unveiled. Gross domestic product (GDP) per capita were found to have an impact on this association. However, even after controlling it, RMC remained significantly associated with cancer incidence (0.463, *p* < 0.001; 0.592, *p* < 0.001). Meanwhile, after controlling GDP per capita, the correlation coefficients between white meat consumption and overall cancer incidence were found to be much lower and insignificant, at 0.089 (*p* = 0.288) for poultry consumption and at −0.055 (*p* = 0.514) for seafood and fish consumption. Notably, an interesting comparison was performed between changes of colorectal cancer incidence and RMC in many countries and regions. A lag of 15–20 years was found, implying causality between RMC and cancer risk. Our findings will contribute to the development of more rational meat consumption concept.

## 1. Introduction

Meat is a crucial component of the human diet, providing essential high-quality protein [1,2]. Given that meat offers appealing flavor and essential nutrients [3,4], its global consumption has increased in tandem with improvements in living conditions [1]. The annual per capita meat consumption has steadily increased from 32.10 kg/year in 1961 to 62.57 kg/year in 2019 (according to Our World in Data). This trend is particularly prominent in developing countries and regions [1], such as China, where the annual per capita meat consumption has soared from a mere 7.62 kg/year in 1961 to 102.17 kg/year in 2019 (according to Our World in Data). Accordingly, to develop a more rational concept of meat consumption, it is pertinent to investigate the potential impact of increased meat consumption on human health.

With the continuous increase, more and more researchers believe that the meat consumption pattern should also receive sufficient attention [1,5], especially its impact on health. Commonly, the meat is classified into two categories: red meat and white meat. However, currently, there is no universal definition to differentiate between the two [4,6,7,8]. Typically, red meat is defined as mammalian-derived meat, including pork, beef and lamb, while white meat refers to non-mammalian sources such as poultry, seafood and fish. 

There is growing evidence suggesting that higher red meat consumption (RMC) may not be beneficial for human health [1,9,10]. Several epidemiological and pathological studies have reported a positive association between RMC and the incidence of cancer [11,12,13,14], while no positive association has been found between the consumption of white meat and cancer incidence [15,16,17,18,19,20,21]. Moreover, the International Agency for Research on Cancer (IARC) classified red meat as a Group 2A carcinogen in 2015 [6].

However, the results remain greatly controversial by now. There are still studies in this field that have failed to establish such a positive association [4,22,23,24,25], though positive associations between RMC and the incidence of various cancers have been reported in some cohort and case–control studies [11,12,13,14]. Additionally, the IARC classification also indicates that the carcinogenicity of red meat remains highly uncertain [6]. The uncertainty severely hampers the development of a more rational meat consumption concept.

The inconsistency of the studies may be attributed to the limitations of sample sizes, low accuracy of consumption assessment methods, and inadequate research duration. Notably, most of the studies relied on self-reported meat consumption data from participants [7,11,12,13,14,26]. To overcome these limitations, meta-analyses have been frequently conducted based on large amounts of data from previous studies [27,28,29,30]. Recently, to obtain more accurate results, in some meta-analyses, a new risk assessment was applied [31,32,33,34,35]. Nevertheless, meta-analyses may introduce potential confounding factors [36], such as geographical differences, GDP per capita, heterogeneity among study designs and differences in population characteristics, and these meta-analyses also failed to reach consistent and definitive conclusions. Consequently, a study with larger, more objective, and longer-term data is highly necessary now to systematically unveil the association between RMC and cancer incidence accurately.

Although epidemiological studies have frequently reported a positive association between RMC and specific cancer incidence, these studies do not mechanistically demonstrate the association. To understand the mechanisms underlying the higher incidence of cancer with higher RMC, some researchers have conducted pathological studies [37,38,39,40]. However, the evidence of the relevant factors’ contribution is not conclusive either. Therefore, a definitive conclusion regarding the positive association between cancer risk and RMC cannot be reached based solely on these findings. 

With the advancement of the internet, numerous global databases have been established and expanded, generating massive amounts of systematically collected data, including consumption and disease incidence [41]. Some of these data have already been utilized for other research analysis, leading to significant findings [42,43,44,45]. This suggests that the global data on RMC and cancer incidence can also be analyzed to make the conclusion clearer. To the best of our knowledge, no such studies have been conducted to date in the area of RMC and cancer risk association exploration.

Consequently, to reveal the definite association between RMC and cancer risk, global consumption data of pork, beef, mutton, poultry, seafood, and fish from Our World in Data and cancer incidence data from the Global Cancer Observatory were obtained and systematically analyzed for the first time here. Correlation coefficients were calculated, and the effects of geographical and economic factors on correlation coefficients were analyzed. Furthermore, changes of RMC and cancer incidence over an extended period were examined. Comparative analysis of white meat was also conducted to understand the association between RMC and cancer risk further. 

## 2. Methods

### 2.1. Data Source and Selection

#### 2.1.1. Association between Meat Consumption and Cancer Incidence

Data on the annual per capita consumption of pork, beef, lamb, and poultry in 182 countries and regions for the period of 1961–2017 were obtained from Our World in Data (https://ourworldindata.org/grapher/per-capita-meat-consumption-by-type-kilograms-per-year (accessed on 1 February 2023)). Similarly, data on annual per capita seafood and fish consumption (SFC) in the same period for 182 countries and regions were obtained from the same source (https://ourworldindata.org/grapher/fish-and-seafood-consumption-per-capita (accessed on 1 February 2023)). It is worth noting that seafood consumption and fish consumption were treated as one item here, encompassing all major seafood categories such as crustaceans, cephalopods, and mollusks, as well as various fish species. Furthermore, data on the annual overall cancer incidence (OCI) from Our World in Data (https://ourworldindata.org/grapher/cancer-incidence (accessed on 1 February 2023)) for the period of 1990–2017, covering 195 countries and regions, were collected.

As for selection, 177 countries and regions with both the OCI and meat consumption data were chosen at first, spanning the years from 1990 to 2017 for OCI data and from 1961 to 2017 for meat consumption data. However, the statistical year of the data is not exactly the same for all countries and regions. In order to balance as many countries and regions as possible and a longer year span, this span was reduced to 1992–2017. At this time, there are still some missing data countries and regions, such as Belgium (its meat consumption data was only available from 2000 to 2017). These are excluded eventually. The final number of retained countries and regions was 159. The annual per capita consumption of pork, beef, and lamb was summed as annual RMC per capita. Finally, RMC, poultry meat consumption (PMC), SFC and OCI data of the each retained countries and regions were averaged. 

The annual incidences of 26 types of cancer (colon, rectum and anus are grouped as colorectum) for males and females across 42 countries and regions, spanning the years from 1943 to 2018, were obtained from the Global Cancer Observatory (https://gco.iarc.fr/overtime/ (accessed on 1 February 2023)). Additionally, gender ratios for these countries and regions from 1960 to 2021 were obtained from The World Bank (https://data.worldbank.org/indicator/SP.POP.TOTL.MA.ZS?end=2021&start=2021&view=map&year=2021 (accessed on 1 February 2023)). These data were utilized to calculate cancer incidence for both genders (excluding breast cancer, prostate cancer, testicular cancer, etc.). Following similar procedure described in the above paragraph, a total of 40 countries and regions spanning from 1999 to 2010 were selected. In addition, data on meat consumption in these countries and regions were collected correspondingly, and these items were summed and averaged as described above.

In order to systematically understand the associations between RMC and cancer incidence by cluster analysis, incidences of 36 types of cancer (colon, rectum and anus are separated) in 185 countries and regions in 2020 were obtained from the Global Cancer Observatory (https://gco.iarc.fr/today/ (accessed on 1 February 2023)).

#### 2.1.2. Effect of Regional Conditions and Customs

To calculate the partial correlation coefficients between OCI and meat consumption, annual gross domestic product (GDP) per capita for the years 1992–2017 was collected from Our World in Data (https://ourworldindata.org/grapher/gdp-per-capita-in-us-dollar-world-bank (accessed on 1 February 2023)) for 144 countries and regions (from the 159 countries and regions), and then averaged. Likewise, to calculate the partial correlation coefficients between colorectal cancer (CRC) incidence and meat consumption, annual GDP per capita for the years 1999–2010 was obtained from the same website for the abovementioned 40 countries and regions, and was also averaged.

#### 2.1.3. Lag of Influence from RMC on CRC Incidence

When investigating the lag of influence from RMC on CRC incidence, separate analyses were performed for each country, and data from 41 countries were used (data from The United Kingdom of Great Britain and Northern Ireland were excluded, as some data were missed). The available annual CRC incidence and annual meat consumption data for all these 41 countries and regions were used without any selection or averaging.

### 2.2. Statistical Analysis

#### 2.2.1. Association between RMC and Cancer Incidence

Since the data were not normally distributed, Spearman correlation coefficient was chosen to determine the correlation. The Spearman correlation coefficients were calculated using SPSS (V 25) to assess the relationship between OCI and RMC, as well as between RMC and the incidences of 26 types of cancer. A significance level of *p* < 0.05 was applied. Scatters and bubble charts were generated using Prism 9.

Cluster analysis of the incidences of 36 cancers in 2020 across 185 countries and regions was performed using Clustvis (https://biit.cs.ut.ee/clustvis/ (accessed on 1 February 2023)). Cancer incidences and countries were clustered vertically and horizontally, respectively. A heatmap was created using the same software. Since the volume of data is large, the impact of noise should be minimized. Therefore, correlation distance measure was used to calculate the distance between rows or columns, and average linkage criterion was used to cluster rows or columns. Moreover, analysis of variance (ANOVA) was performed to validate the clusters (Table S2).

#### 2.2.2. Effect of Regional Conditions and Customs

Geographic heat maps of RMC, OCI, and GDP per capita (for 144 countries and regions) were created using Tableau Desktop 2022.2 to visually compare the data. To account for the potential influence of GDP per capita on the relationship between RMC and cancer incidence, partial correlation coefficients were calculated using SPSS (V 25) with GDP per capita as the control variable. The first-order partial correlation coefficients between OCI and RMC (n = 144) and between CRC incidence and RMC (n = 40) were computed.

#### 2.2.3. Lag of Influence from RMC on CRC Incidence

The changes in RMC and CRC incidence were compared for each of the 41 countries and regions. Line charts were drawn using Prism 9 to visualize RMC changes and CRC incidence changes in these countries and regions. Based on the analysis of these countries and regions, the lag of influence from RMC on CRC incidence was estimated in detail.

#### 2.2.4. Association between Poultry Meat Consumption or SFC and Cancer Incidence

Spearman correlation coefficients between the OCI (averages from 1992–2017, n = 159) and PMC or SFC, as well as between the average CRC incidence (averages from 1999–2010, n = 40) and PMC or SFC, were calculated. Additionally, partial correlation coefficients between OCI and PMC or SFC (n = 144) were calculated, with annual GDP per capita (averages from 1992–2017) as the control variable. Similarly, partial correlation coefficients between CRC incidence and PMC or SFC (n = 40) were calculated, with annual GDP per capita (averages from 1999–2010) as the control variable.

## 3. Results and Discussion

### 3.1. RMC and Cancer Incidence

#### 3.1.1. Association between RMC and OCI

**Figure 1:**
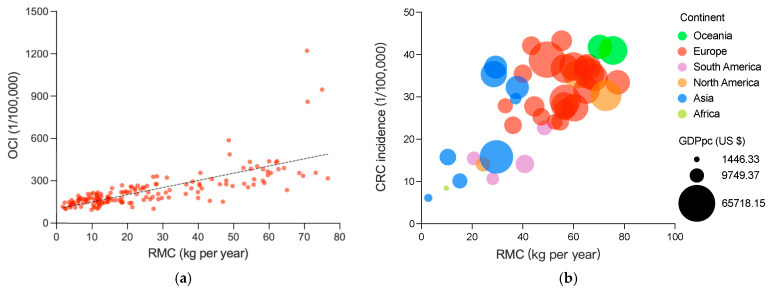
Associations between RMC and OCI or CRC incidence: (**a**) the association between RMC and OCI, RMC and OCI are averages from 1992 to 2017, including 159 countries and regions; and the (**b**) association between RMC and CRC incidence; bubble colors represent the different continents and bubble size represents the GDP per capita. RMC and CRI are averages from 1999 to 2010, including 40 countries and regions. The results of the dose–response analysis is shown in Figure S1.

Figure 1a shows a significantly positive association between RMC and OCI, with a correlation coefficient of 0.798 (*p* < 0.001), which indicates a significant and strongly positive association between the two variables. After being tested, this result is reliable (Table S1). Previous studies have largely focused on the association between specific cancer incidence and RMC, rather than on the association between OCI and RMC. Only a few studies covered overall cancers, and the results were inconsistent [46,47]. By analyzing much larger and more objective data, this study has allowed us to draw the conclusion that there is a strongly positive association between OCI and RMC.

According to previous studies on specific cancer incidence [4,22,23], due to a limited sample size, the conclusion regarding the association between RMC and cancer incidence remains uncertain. In previous cohort studies, the sample size was often less than 100,000 participants; the insufficient sample size would limit the certainty of conclusion drawing. To address this limitation, larger data were collected, including a 470,000 person cohort study [14]. Additionally, meta-analyses were conducted [27,28,29,30,31], most of which covered between 1 million to 10 million participants. By contrast, the significance of this work is highlighted by the involvement of at least 5 billion people (estimated from population data from 159 countries and regions in 1992). With such a large sample size, the certainty of the conclusions is greatly improved. 

In addition, epidemiological studies commonly relied on self-assessment by participants to obtain RMC data [7,11,12,13,14,26], which raised questions about the objectivity and accuracy of the data. The systematic reviews also depend on those data. In contrast, the global data in this study, regardless of the meat consumptions or the cancer incidences, were all obtained from third-party databases. These data were obtained from credible sources with careful collection and strict review processes. In the process of data collection and updating, the regulation is standard, consistent and open. More importantly, data collection is not based on any research purposes. So, it is believed to improve the objectivity and accuracy. 

However, the data were obtained passively by us, which may lead to data incompleteness. In order to minimize these issues, the missing data countries and regions were excluded, and an appropriate time span was fixed to guarantee the maximum volume of data. After exclusion and time span setting, the data still cover 159 countries and regions, and have a span of 26 years (for details, see Section 2.1.1).

#### 3.1.2. Association between RMC and CRC Incidence

Association between RMC and incidence of CRC is a popular but still disputed topic [1,14,22,30,48], thus it was calculated here to further confirm the association (Figure 1b). The correlation coefficient of 0.625 (*p* < 0.001) again suggests a statistically significant and strongly positive association between RMC and CRC incidence. 

Regarding specific types of cancer, CRC is one of the most commonly reported cancers that frequently shows a positive association with RMC [1,2,10,14,27,30,31]. Despite these studies, the association between CRC incidence and RMC also remains a controversial issue [4,22]. In this study, we focused specifically on examining this relationship and found that our results align with most cohort studies and meta-analyses. The findings provide further evidence supporting the positive association between CRC incidence and RMC. Consequently, the above-mentioned controversy is addressed to some extent.

#### 3.1.3. Association between RMC and Other Specific Cancer Incidences

In Table 1, the correlation coefficients between incidences of 26 different other cancers and RMC were presented. The range of correlation coefficients was wide, spanning from −0.473 (*p* = 0.002) to 0.771 (*p* < 0.001). Interestingly, several unexpected or counterintuitive results were observed. For instance, the incidences of stomach and cervix uteri cancer were found to be negatively associated with RMC, with correlation coefficients of −0.392 (*p* = 0.012) and −0.473 (*p* = 0.002), respectively.

Significant and positive associations have been observed between RMC and the incidences of 17 types of cancer. Notably, positive associations have been found between RMC and the incidences of nine cancers, such as skin melanoma, multiple melanoma, leukemia, testis cancer, Hodgkin lymphoma, brain (CNS) cancer, corpus uteri cancer, ovary cancer and oropharynx cancer. However, to the best of our knowledge, previous studies have not given sufficient attention to the associations between these nine cancer incidences and RMC.

Although the association between CRC incidence and RMC is the most commonly reported one previously, and the positive association has been confirmed in Section 3.1.2, it is noteworthy that the correlation coefficient between CRC incidence and RMC is not the highest among the 26 cancers studied (Table 1). Specifically, we identified five cancer types (multiple myeloma, skin melanoma, breast, leukemia, and prostate) with positive correlation coefficients higher than that of CRC incidence. It is worth to mention that the associations between RMC and incidences of multiple myeloma, skin melanoma and leukemia have not been adequately explored.

The associations between RMC and the incidences of stomach and cervix uteri cancers were unexpected and surprising, which differed from those of other cancer types studied. A meaningful negative association between stomach cancer incidence and RMC was observed (−0.392, *p* = 0.012), contrary to previous reports [28], suggesting a positive association. Meanwhile, the identified negative association between RMC and cervix uteri cancer incidence (−0.473, *p* = 0.002) was unreported by now.

These findings highlight the need for further investigation into the association between RMC and specific cancer incidence, particularly for the newly identified associations reported here, and further research is needed to elucidate the underlying mechanisms and potential implications of these unexpected findings.

### 3.2. Effect of Regional Conditions and Customs

#### 3.2.1. Cluster Analysis of Countries and Regions

**Figure 2:**
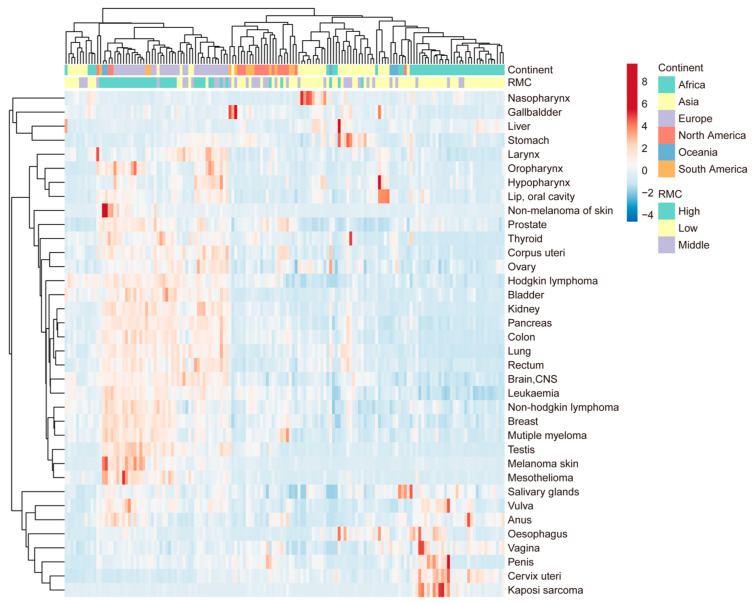
Associations between RMC and specific cancer incidences: cluster analysis of 36 cancer types; cancer incidence is for the year 2020, including 153 countries and regions. The average RMC from 1992 to 2017 is used to assess the level of RMC in these areas, with 0–20 kg per year defined as low, 20–40 kg per year defined as middle, and more than 40 kg per year defined as high.

To systematically investigate the associations between RMC and cancer incidence, a cluster analysis was performed (presented in Figure 2). The 153 countries and regions were clustered into two major groups, with the larger group was further subdivided into three clusters. Coincidentally, these four groups correspond to the four continents (Europe, Asia, Africa, and America). The incidences of 36 cancer types were clustered into three major groups. As illustrated in Figure 2, the three cancer clusters were found to be highly prevalent in Europe (with high RMC), Asia (with middle RMC), and Africa (with low RMC), respectively. However, special cancer incidence profile was not found in South America and North America (with differing RMC).

The findings from both the clustering analysis and correlation analysis exhibited a remarkable concordance. Specifically, it is found that the cluster of cancers with high prevalence in Europe (with high RMC) includes all cancers that have correlation coefficients higher than 0.3 in Table 1. Furthermore, the cancers with insignificant or negative coefficients in Table 1 were clustered into the other two groups, namely Africa with low RMC, and Asia with middle RMC. In Figure 2, it was observed that cervix uteri, vulva, anus, salivary gland, penis, vagina and oesophagus cancer and Kaposi sarcoma (low correlation coefficients) were clustered into the same group (Africa with low RMC).

Concerning the cluster analysis results, these four divided groups of countries and regions roughly coincide with four continents. Additionally, most cancers with low coefficients (cervix uteri, vulva, anus, salivary gland, penis, vagina and oesophagus cancer and Kaposi sarcoma) were prevalent in the Africa group (with low RMC). It should be noted that the great majority of these cancers are associated with viral infections, such as HPV or HIV [49,50]. This cannot help but remind us of the impact of geographical factors on the association.

#### 3.2.2. Distribution and Partial Correlation Analysis of GDP per Capita

**Figure 3:**
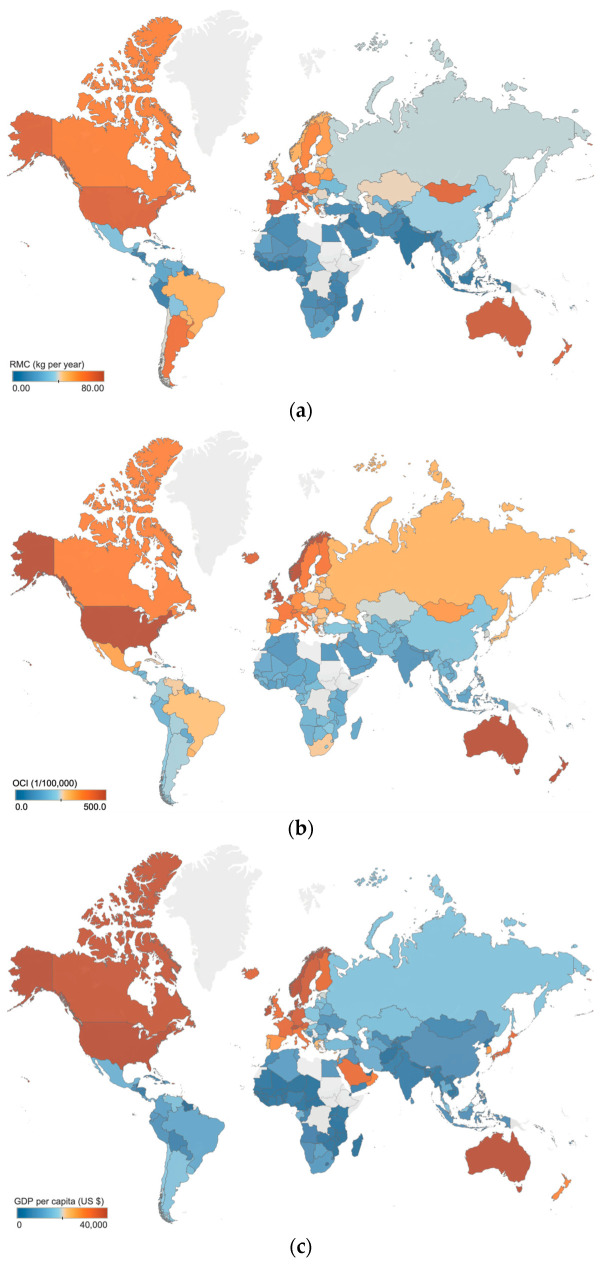
Distribution of RMC, OCI and GDP per capita: (**a**) the distribution of RMC; (**b**) the distribution of OCI; (**c**) the distribution of GDP per capita. The averaged data from 1992 to 2017 are used to assess the level of local RMC, OCI and GDP per capita, including 144 countries and regions. The gray areas in the maps indicate no data.

In Figure 3, the distribution of RMC, OCI, and GDP per capita across different regions is shown. The data demonstrate that these three variables were all the highest in Europe, followed by Asia, and the lowest in Africa. Furthermore, countries and regions located in the same continent were found to exhibit similar RMC and cancer incidence. It is important to acknowledge how the regional conditions and cultural practices potentially influence the association between RMC and cancer incidence [36], and which factors are crucial to be taken into consideration. It remains unknown whether some factors, such as geography and GDP per capita, could significantly confound the conclusion drawing. It has been widely observed that regions with higher GDP per capita tend to exhibit higher RMC and cancer incidence [1,51]. Therefore, among the various factors that influence these trends, GDP per capita is probably the most influential and comprehensive.

To exclude the influence of GDP per capita, the partial correlation coefficient between RMC and OCI was calculated (the linearity assumptions are met, as shown in Figure S2). The value was 0.463 (*p* < 0.001), indicating a moderately positive and still significant association. Similarly, we also calculated the partial-correlation coefficient between RMC and CRC incidence. The value (0.592, *p* < 0.001) also indicated a significant and moderately positive association, although weaker than the former. In a word, both of these associations remain significant and moderately positive, even after accounting for the influence of GDP per capita. 

The possible influence of regional conditions and customs has been pointed out in previous meta-analyses [36]. However, due to the lack of systematic data, this impact has rarely been systematically and quantitatively discussed, and it may further contribute to the uncertainty of the previous conclusions [36]. It is also clear from these results that the correlations are indeed affected by GDP per capita, and all of them decrease after controlling for GDP per capita. Moreover, it is confirmed that although GDP per capita does have an impact, after controlling it, the positive partial correlation coefficient between RMC and OCI or CRC incidence remains significant.

### 3.3. Lag of RMC’s Influence on CRC Incidence 

It is important to note that a high and significant correlation coefficient between two sets of data does not necessarily prove a direct cause-and-effect association between them [52]. So far, in all those correlation analyses, no one has definitively stated that increased RMC or GDP is a cause of increased cancer risk, although the positive correlation coefficients have frequently been obtained.

**Figure 4:**
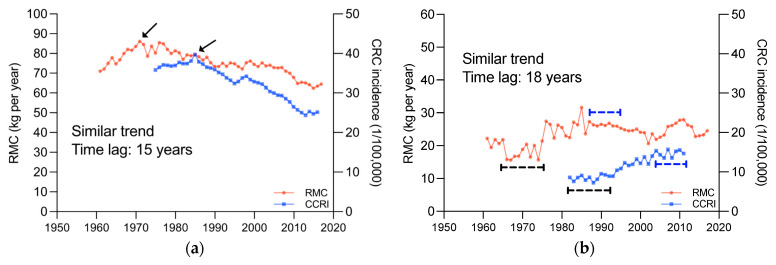

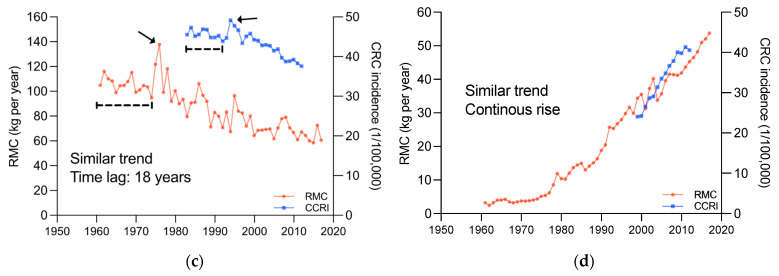
Changes in RMC and CRC incidence, 1961~2017; (**a**), United States of America. (**b**), Costa Rica. (**c**), New Zealand. (**d**), Republic of Korea. The area indicated by the arrows and line segments is the basis for determining similarity.

As such, the influence of time dimension on the association between RMC and cancer incidence are also worth to be explored. The changes of RMC and CRC incidence in 41 countries and regions were compared (Figure 4, more details see also Figures S3 and S4). The results indicated that in most countries and regions, CRC incidence changes exhibited trends similar to RMC changes, and lagged behind RMC changes by about 15–20 years. 

The RMC and CRC incidence changes in four typical countries, United States of America, Costa Rica, New Zealand, and Republic of Korea, were displayed in Figure 4. In United States of America (Figure 4a), RMC showed an upward and then downward trend, peaking in 1970, and CRC incidence also showed an upward and then downward trend, peaking in 1985, with similar trends and a lag of around 15 years. In Costa Rica (Figure 4b), both RMC and CRC incidence exhibited “S”-shaped trends, with a lag of around 18 years. In New Zealand (Figure 4c), both RMC and CRC incidence showed an overall downward trend, but with an abrupt upward trend in 1974 and 1992, respectively, and there was a lag of around 18 years. In Republic of Korea (Figure 4d), both RMC and CRC incidence were continuously increasing, without any noticeable changes in trend. This phenomenon has not been reported before.

This novel finding sheds new light on the relationship between these two factors. The similar trends and noticeable lag strongly imply that CRC incidence changes may be caused by RMC changes. Moreover, it is also suggested that the change in CRC incidence caused by the change in RMC may not be observable sufficiently since epidemiological studies are limited by the short follow-up duration; namely, many studies on humans have lasted much less than 10 years [12,13,14,17]. Therefore, we reasoned that the uncertainty of causality from such studies may be related to the short duration.

### 3.4. Association between PMC or SFC and Cancer Incidence

**Figure 5:**
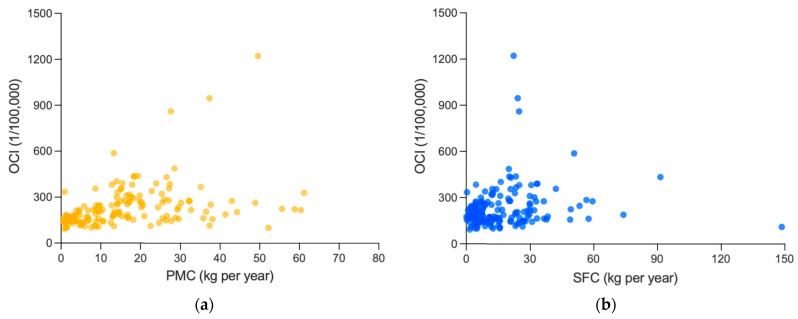

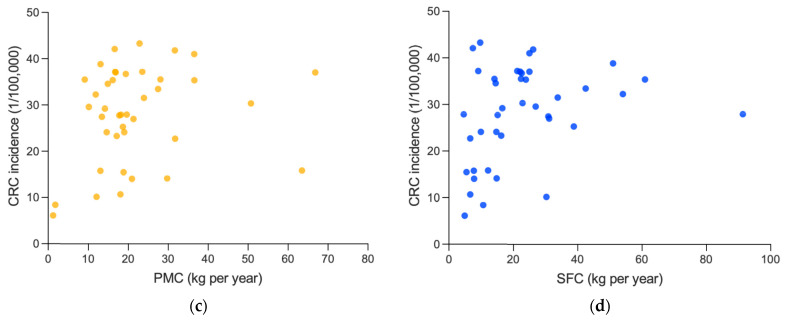
Association between white meat consumption and cancer incidences: (**a**) association between PMC and OCI; and (**b**) association between SFC and OCI. PMC, SFC and OCI are averages from 1992 to 2017, including 159 countries and regions. (**c**) Association between PMC and CRC incidence. (**d**) Association between PMC and CRC incidence. PMC, SFC and OCI are averages from 1999 to 2010, including 40 countries and regions.

To facilitate comparative analysis with RMC, the association between white meat consumption and cancer incidence was explored (as shown in Table 2 and Figure 5). It is worth noting that PMC, and SFC were reported separately in most studies [15,16,17,18,19,20,21,28]. In this analysis, the convention was followed.

At first, the analysis revealed that there is a significant and moderately positive association between PMC and OCI (0.499, *p* < 0.001), and a significant and weakly positive correlation coefficient between SFC and OCI (0.213, *p* = 0.007), which were both weaker than that of RMC (0.798, *p* < 0.001). Similar results were obtained in CRC incidence. The correlation coefficient of RMC (0.625, *p* < 0.001) here was also more significant and stronger than those of PMC (0.232, *p* = 0.150) or SFC (0.330, *p* = 0.038).

However, referring to Section 3.2.2, after controlling GDP per capita, both PMC (0.089, *p* = 0.288) and SFC (−0.055, *p* = 0.514) were non-significantly associated with OCI, different from the remained significantly positive association between RMC and OCI (0.463, *p* < 0.001), and the partial correlation coefficients between PMC (−0.018, *p* = 0.912) or SFC (0.081, *p* = 0.625) and CRC incidence were also non-significant, and much weaker than that between RMC and CRC incidence (0.592, *p* < 0.001). Thus, the positive association of PMC or SFC mentioned above might probably be attributed to the influence of GDP per capita.

In a word, after taking into account GDP per capita, the two associations are almost negligible, and the P values are all higher than 0.05, consistent with previous epidemiological studies [15,16,17,19,28]. Some studies even suggest a negative relationship between SFC and cancer incidence [18,20,21]; however, it is not seen here. Thus, the positive association between RMC and cancer incidence is more conclusive after comparison with the association between white meat consumption and cancer incidence.

To further fix the effect of GDP per capita, case analysis was performed, as presented in Table 3. In the table, data of PMC, SFC, RMC, OCI and GDP per capita of the top 22 countries and regions with the highest GDP per capita and regions in this study was selected from the 159 countries. Notably, the RMC in Kuwait, Japan, Saudi Arabia, and Oman are found to be distinctly lower from other countries and regions, but the PMC or SFC of them are higher. Consistent with their lower RMC, the OCI therein were also significantly lower than those in other countries and regions with high RMC. It again proves that it is not PMC or SFC, but RMC positively associated with OCI.

### 3.5. Analysis of Causality between RMC and Cancer Risk

It has been 8 years since the IARC classified red meat as a group 2A carcinogen in 2015 [6], but it seems to have had little impact on meat consumption pattern. In many developing countries, an increase in total meat consumption is still considered as an important indicator of living improvement standards, and the meat consumption is continuously growing [1]. Additionally, meat consumption remains consistently high in developed countries [1]. The concept of a rational meat consumption pattern has attracted little attention. 

The reason for this is probably that the causality between RMC and cancer risk has not been established. Although it cannot definitively determine a causality just from the statistical evidence of correlation found in this research yet; the finding of the lag between CRC incidence and RMC strongly supports that the causality exists. After the associations between PMC or SFC and cancer incidence were analyzed, the comparative analysis results further confirm that higher RMC, rather than other meat consumptions, is associated with higher cancer incidence. Therefore, under the trend of meat consumption rising, more attention should be paid to the rationality of the meat consumption pattern [3,53,54,55].

## 4. Conclusions

In this study, using global data, it systematically confirms that higher RMC is associated with higher cancer incidence. Although the GDP per capita were found to have a great influence on association between RMC and cancer incidence, the significantly positive association remains after controlling this factor. Meanwhile, no association between white meat consumption and cancer incidence was found comparatively after controlling GDP per capita. In addition, some interesting findings in detail were revealed also. Firstly, RMC is significantly and positively associated with OCI and 17 types of cancer incidences. Some of these cancers, such as multiple myeloma, leukemia, testis cancer, have not received adequate attention and require further investigation. Moreover, a lag of around 15–20 years was found between CRC incidence changes and RMC changes. The finding points out the certain causality, and suggests that the follow-up time length in epidemiological studies of the association between RMC and cancer risk should be prolonged enough.

The conclusions based on such a huge, objective third-party database were considered to be reliable, and important in settling the controversy over the association between RMC and cancer risk. Also, they provide directions for future studies on the association between them, and consequently contribute to the development of more rational meat consumption concept, which in turn guides the public towards healthier meat consumption. 

## Figures and Tables

**Table 1:** Correlation coefficients between incidences of cancers and RMC.

Cancer Type	Correlation Coefficient	*p*-Value
Skin melanoma **	0.771	0.000
Multiple myeloma **	0.766	0.000
Breast	0.755	0.000
Leukemia **	0.712	0.000
Prostate	0.682	0.000
Colorectum	0.625	0.000
Testis **	0.608	0.000
Hodgkin lymphoma **	0.562	0.000
Non-Hodgkin lymphoma	0.550	0.000
Bladder	0.525	0.001
Kidney	0.506	0.001
Brain, CNS **	0.470	0.002
Corpus uteri **	0.440	0.004
Lung	0.387	0.014
Ovary **	0.372	0.018
Pancreas	0.361	0.022
Oropharynx **	0.333	0.036
Larynx	0.147	0.364
Oesophagus	0.147	0.367
Kaposi sarcoma	−0.003	0.985
Thyroid	0.019	0.908
Gallbladder	−0.115	0.479
Liver	−0.143	0.379
Uterus	−0.216	0.180
Stomach	−0.392	0.012
Cervix uteri **	−0.473	0.002

Incidences of the 26 cancers and RMC are averages from 1999 to 2010, including 40 countries and regions. A “**” indicates that this type of cancer has not been given sufficient attention previously.

**Table 2:** Correlation coefficients between meat consumption and cancer incidences.

Type	Correlation Coefficient (n = 159/40)	*p*-Value	Partial-Correlation Coefficient (n = 144/40)	*p*-Value
OCI/RMC	0.798	0.000	0.463	0.000
OCI/PMC	0.499	0.000	0.089	0.288
OCI/SFC	0.213	0.007	−0.055	0.514
CRC incidence/RMC	0.625	0.000	0.592	0.000
CRC incidence/PMC	0.232	0.150	−0.018	0.912
CRC incidence/SFC	0.330	0.038	0.081	0.625

The correlation coefficients between OCI and RMC cover 159 countries and the partial correlation coefficients cover 144 countries. The correlation coefficients and partial correlation coefficients between CRC incidence and RMC both cover 40 countries.

**Table 3:** GDP per capita, OCI, RMC, PMC, SFC in high GDP per capita countries and regions.

Countries and Regions	GDP per Capita (US $)	OCI (1/100,000)	RMC (kg/Year)	PMC (kg/Year)	SFC (kg/Year)
NOR	63,400.99	587.52	48.68	13.30	50.83
**KWT**	**52,182.33**	**100.45**	**27.39**	**52.25**	**12.69**
CHE	48,385.96	402.52	56.72	13.98	16.26
USA	46,827.12	1221.51	70.77	49.64	22.37
IRL	41,997.98	430.41	62.03	26.58	21.06
AUS	40,018.90	945.43	75.07	37.40	24.31
DNK	39,982.15	438.24	62.35	19.42	23.40
NLD	39,166.50	437.71	60.06	18.38	20.28
CAN	38,243.56	365.55	59.64	35.24	23.43
SWE	36,893.81	382.05	58.34	12.83	30.59
DEU	36,474.24	354.93	68.56	15.13	14.16
AUT	36,453.57	316.46	76.62	17.21	12.34
ISL	34,852.42	433.72	54.64	18.35	91.45
**JPN**	**34,202.50**	**275.94**	**28.22**	**16.14**	**59.50**
FRA	34,012.14	390.28	64.26	23.94	33.15
FIN	33,659.75	390.65	52.82	15.48	33.59
**SAU**	**33,268.51**	**114.91**	**10.78**	**37.43**	**8.85**
GBR	33,105.29	486.56	48.93	28.57	20.11
ITA	32,559.55	381.81	62.69	17.93	24.96
TWN	30,874.74	274.60	46.16	32.41	32.90
**OMN**	**29,910.23**	**111.72**	**21.39**	**22.54**	**26.51**
NZL	28,864.18	860.11	70.95	27.66	24.95

The GDP per capita, OCI, RMC, PMC, SFC of 159 countries and regions has been arranged based on their GDP per capita (as shown in Table S3), and the top 22 countries and regions have been selected and listed, with their names represented by abbreviations. The GDP per capita, OCI, RMC, PMC, SFC are all averages from 1992 to 2017. Kuwait, Japan, Saudi Arabia, Oman are the four countries that display unique data, with considerably lower RMC compared to other countries that have similar GDP per capita. The data from these four countries are bolded.

## Data Availability

All the data involved in this research are available in the links provided in Methods.

## Supplementary Materials

The following supporting information can be downloaded at: https://www.mdpi.com/article/10.3390/foods12224164/s1.

## Abbreviations

RMC	Red Meat Consumption
PMC	Poultry Meat Consumption
SFC	Seafood and Fish Consumption
OCI	Overall Cancer Incidence
CRC	Colorectal Cancer
GDP	Gross Domestic Product
IARC	International Agency for Research on Cancer
