# Supplementary material for: Red Meat Consumption and Cancer Risk: A Systematic Analysis of Global Data
Source: Foods. 2023 Nov 17;12(22):4164. doi: 10.3390/foods12224164 (PMC10670314; doi:10.3390/foods12224164)
Supplement: Supplementary file 1 [file foods-12-04164-s001.zip › foods-2687264-supplementary.pdf]

Supplementary Material

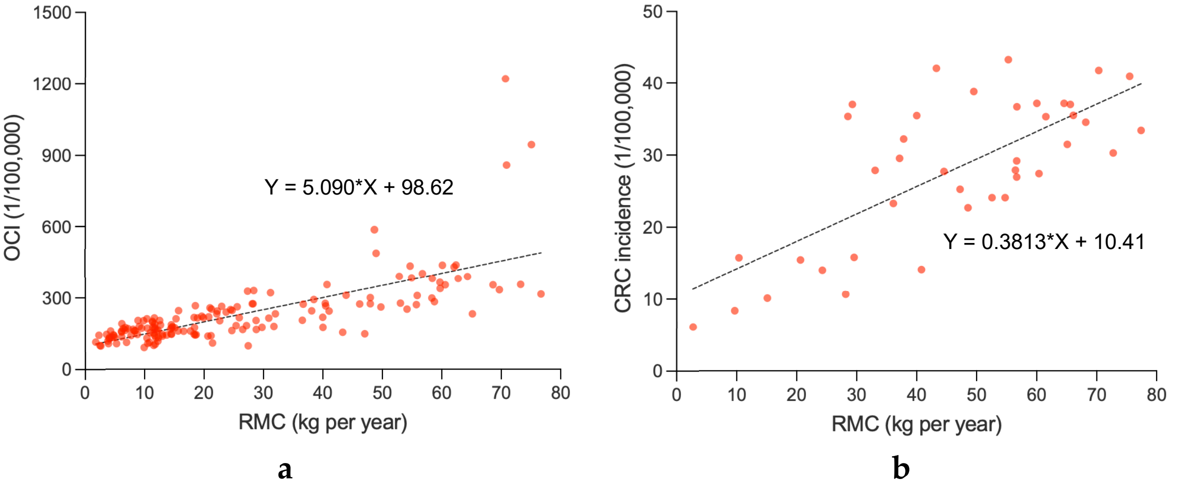

Figure S1. Associations between RMC and OCI or CRC incidence; **a**, Association between RMC and OCI (averages from 1992-2017, n=159). When RMC is very low, the OCI is about 98.62/100,000, and each 10 kg/year increase in RMC may result in an increase in OCI of 50.90/100,000. **b**, Association between RMC and CRC incidence (averages from 1999-2010, n=40). When RMC is very low, the CRC incidence is about 10.41/100,000, and each 10 kg/year increase in RMC may result in an increase in CRC incidence of 3.813/100,000. However, since the data are averaged over many years, the dose-response results may be less applicable to the present.

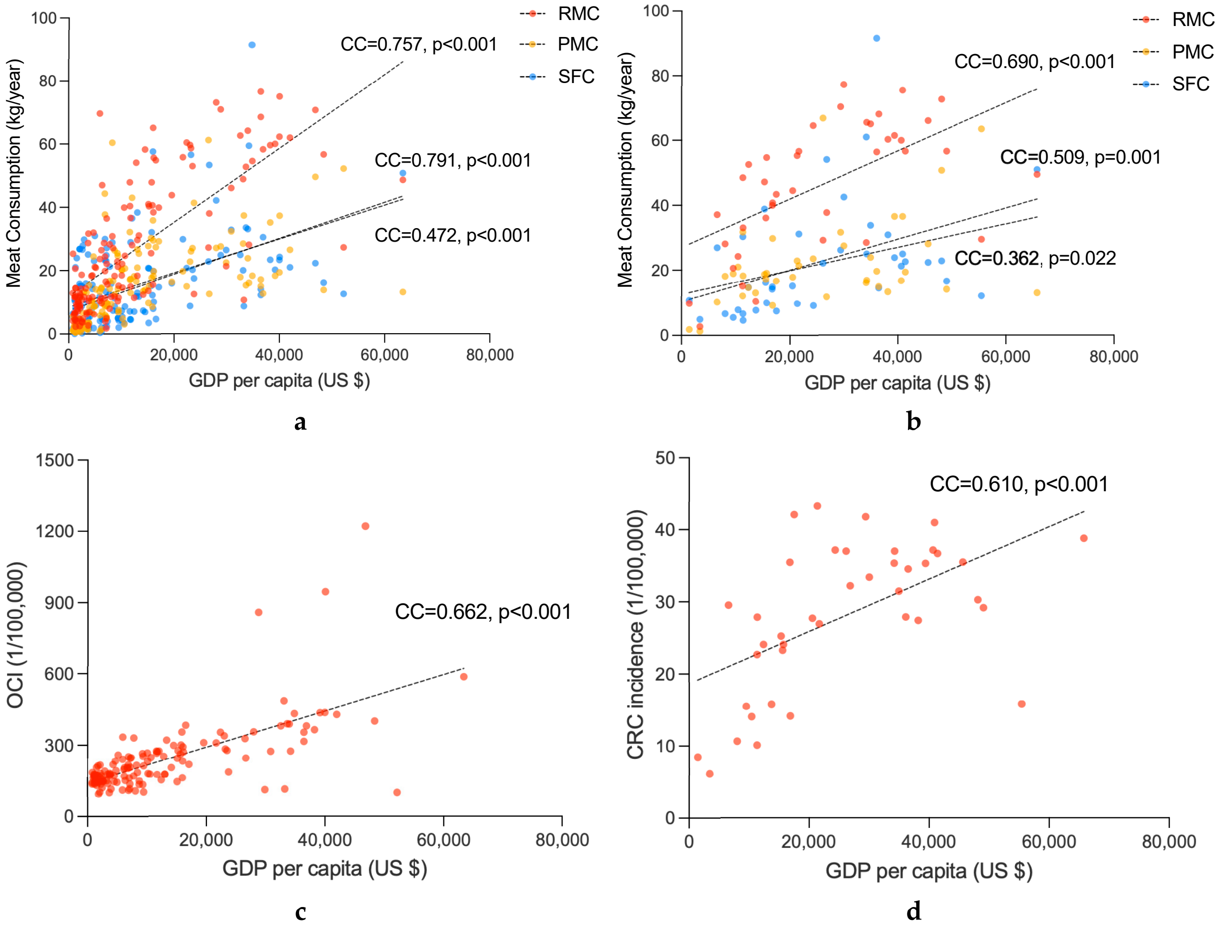

Figure S2. Association between GDP and meat consumptions or cancer incidences; **a**, Association between GDP per capita and RMC, PMC or SFC (averages from 1992-2017, n=144). **b**, Association between GDP per capita and RMC, PMC or SFC (averages from 1999-2010, n=40). **c**, Association between GDP per capita and OCI

(averages from 1992-2017, n=144). **d**, Association between GDP per capita and CRC incidence (averages from 1999-2010, n=40). The GDP per capita are positively and significantly associated with all the items.

Supplementary data for Figure 4 in the main text are listed in the following Figure S3 and Figure S4, where the countries and regions were divided into two groups based on whether their maximum annual per capita RMC exceeds 60 kg per year.

As can be seen from Figure S3 and Figure S4, about half of the countries and regions show similar trends in RMC and CRC incidence, and lag times between RMC and CRC incidence changes can be found in some cases. Moreover, higher CRC incidences generally exist in the countries and regions with higher RMC. However, there are also some countries not in line with this law. In these cases, maybe other factors exerted stronger impacts on the changes in CRC incidence.

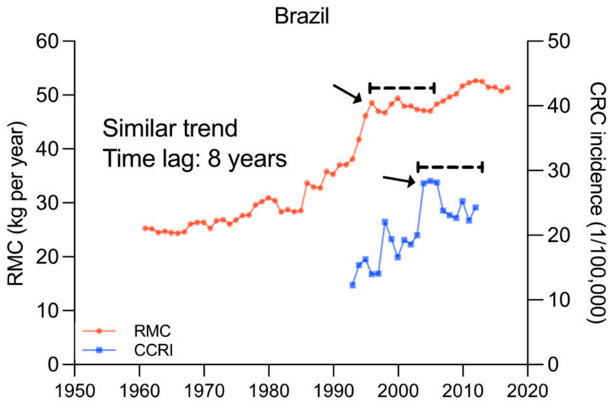

I

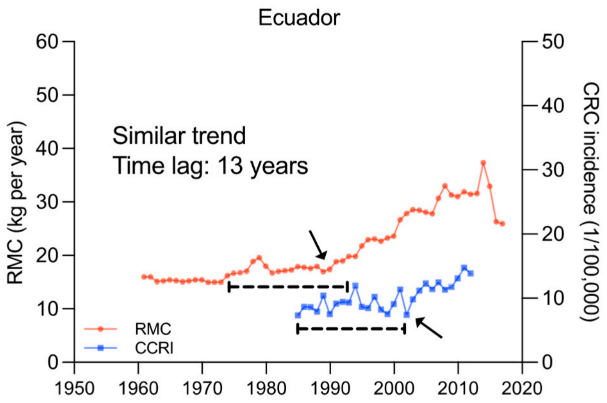

II

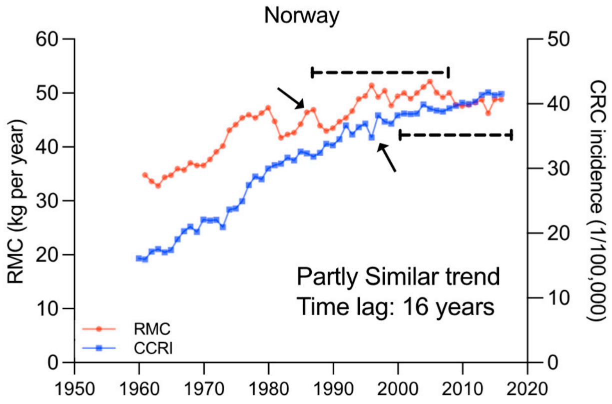

III

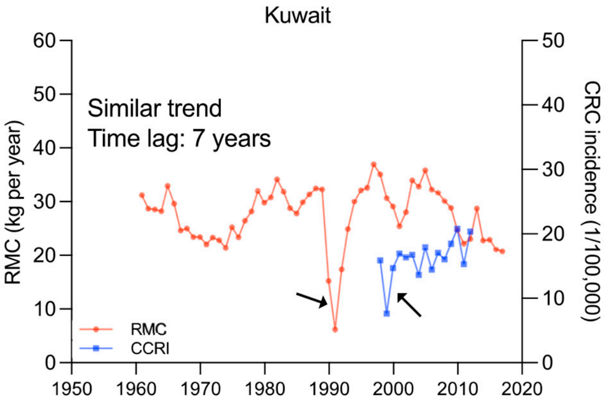

IV

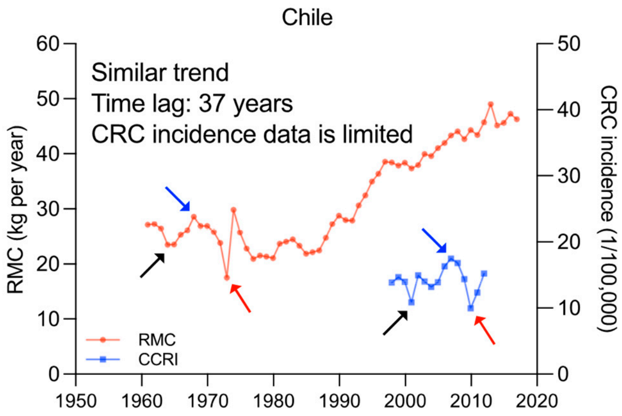

V

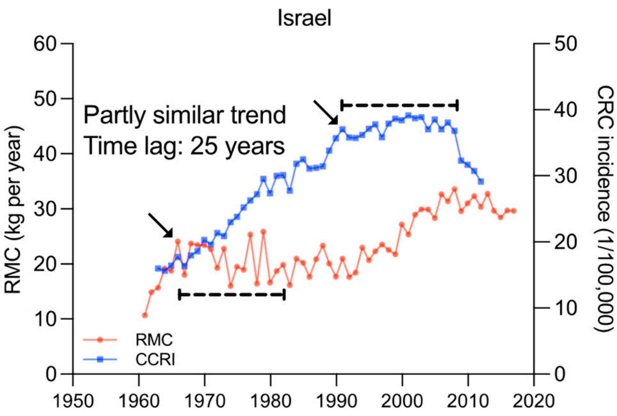

VI

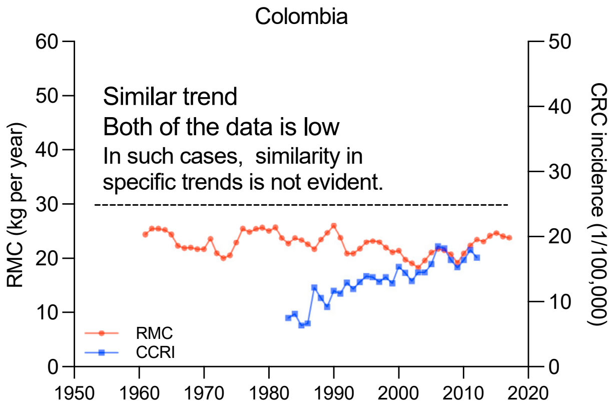

VII

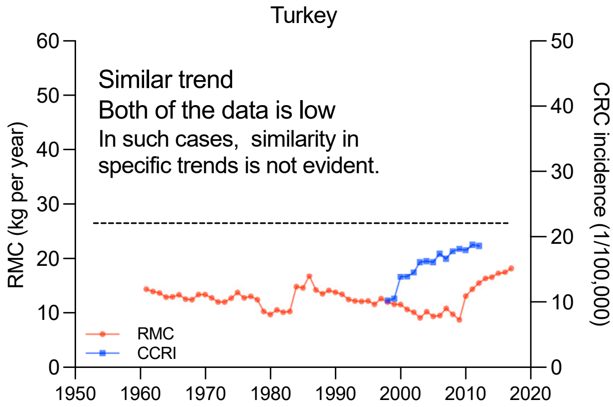

VIII

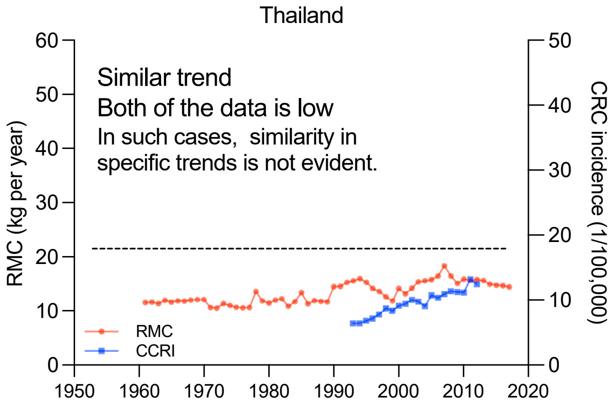

IX

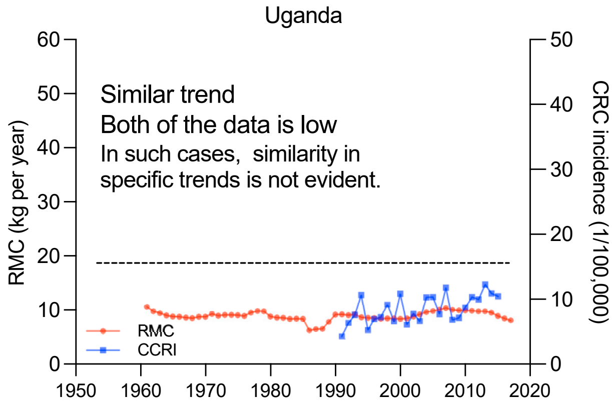

X

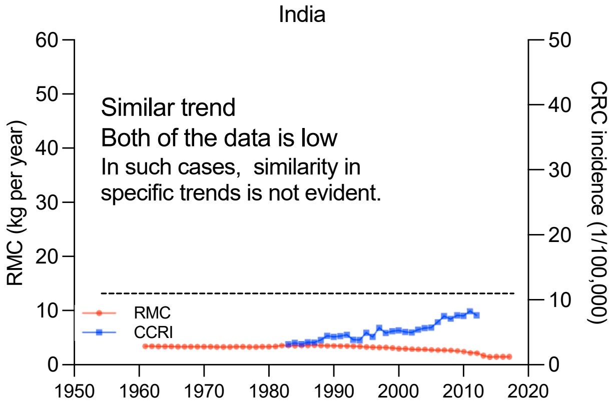

XI

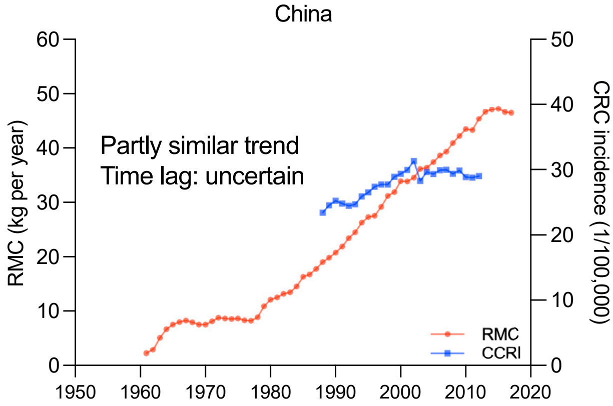

XII

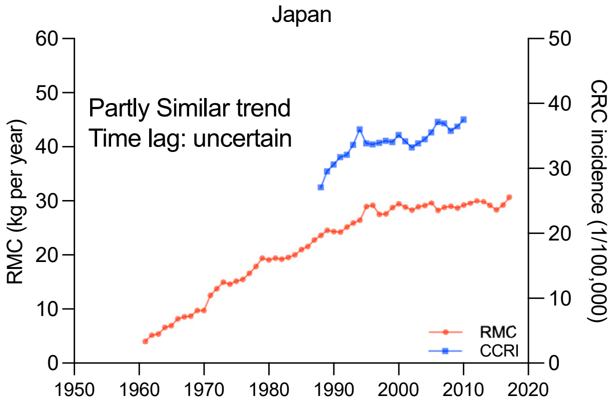

XIII

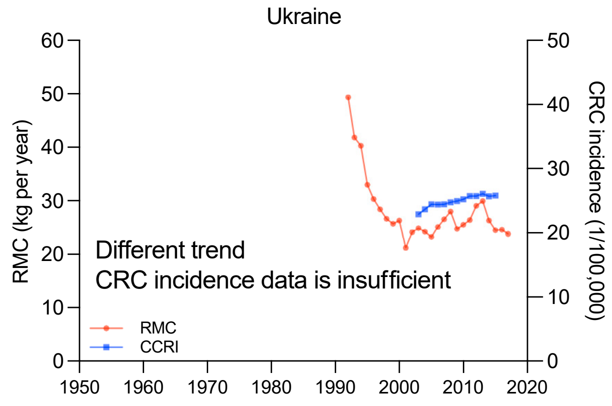

XIV

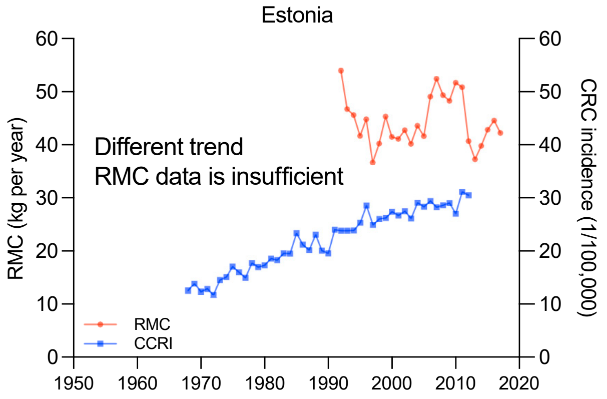

XV

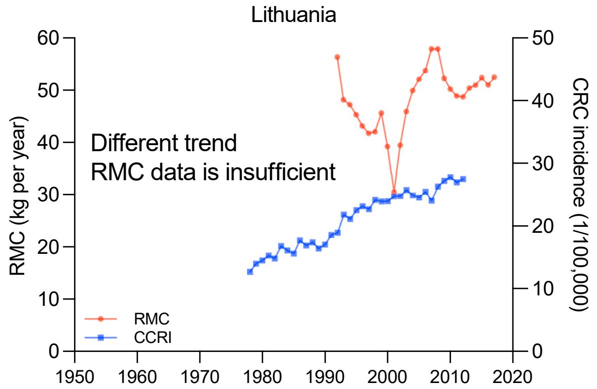

XVI

Figure S3. Time Association between RMC changes and CRC incidence Changes in countries and regions with maximum annual per capita RMC lower than 60 kg per year.

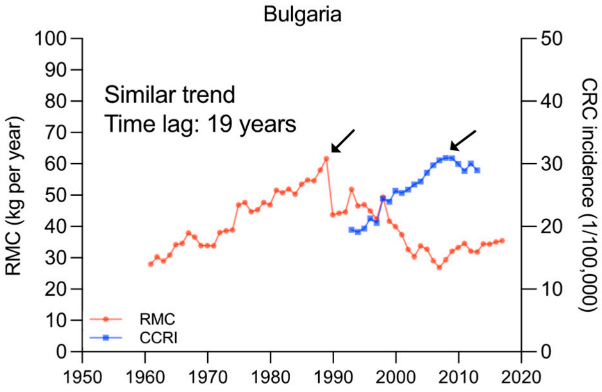

I

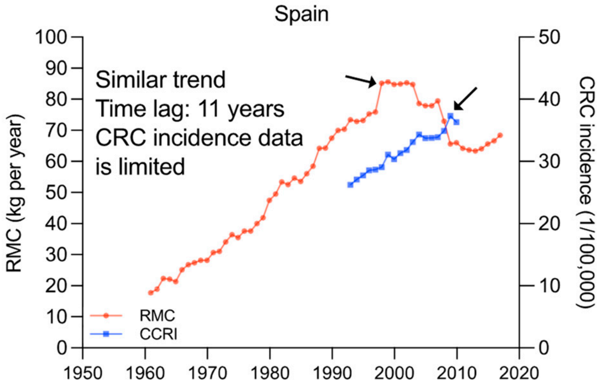

II

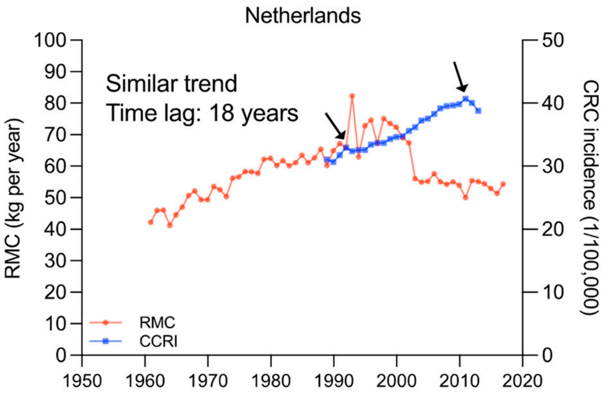

III

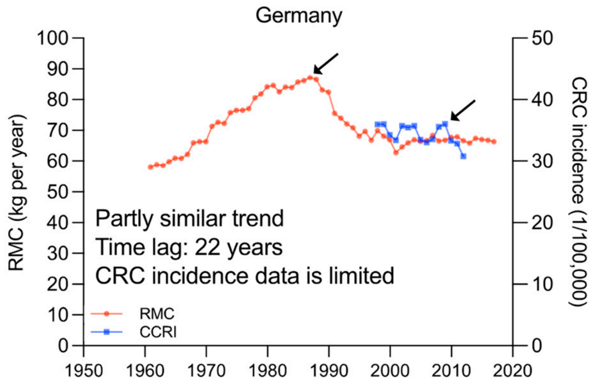

IV

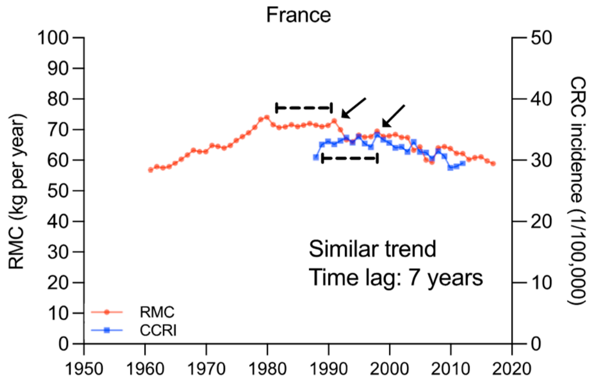

V

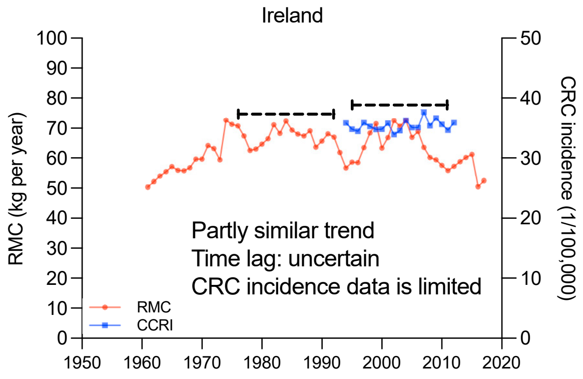

VI

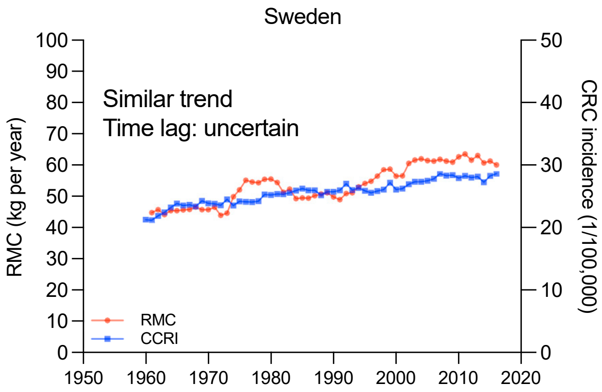

VII

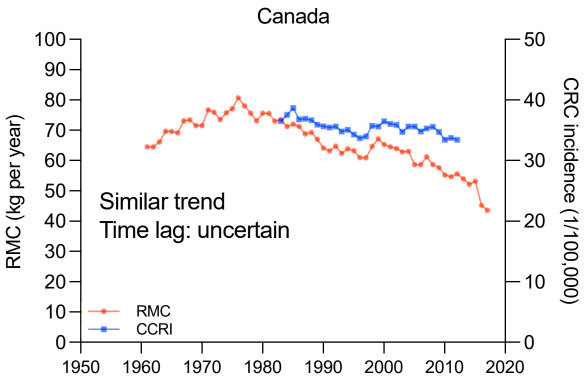

VIII

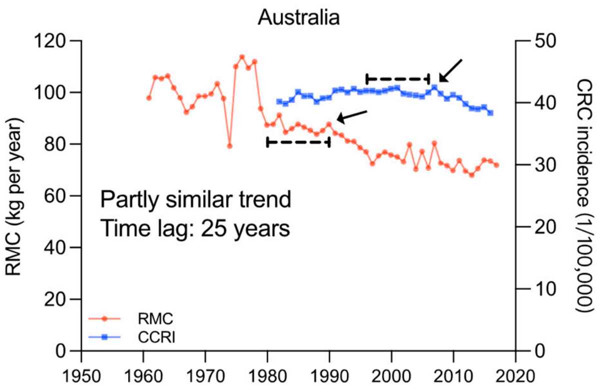

IX

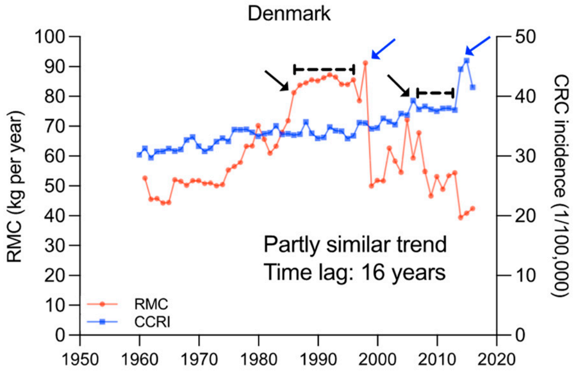

X

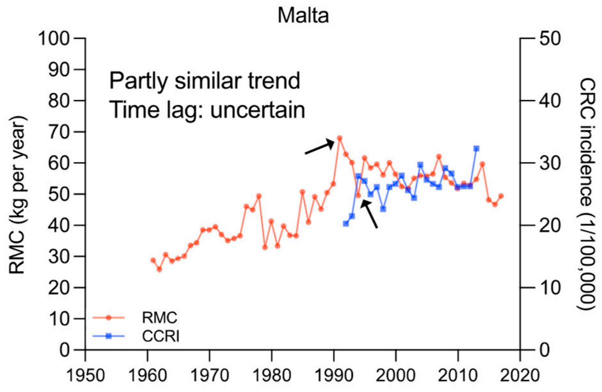

XI

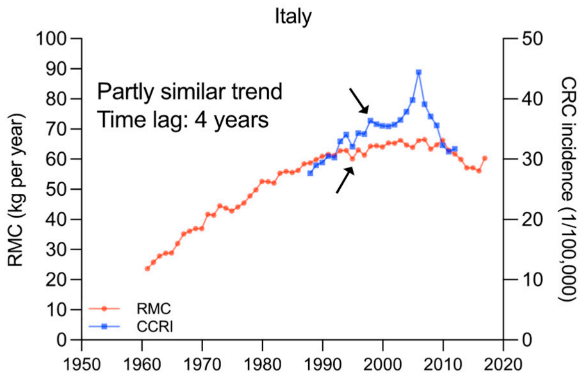

XII

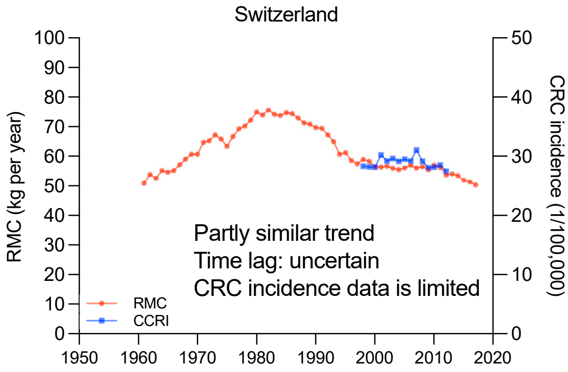

XIII

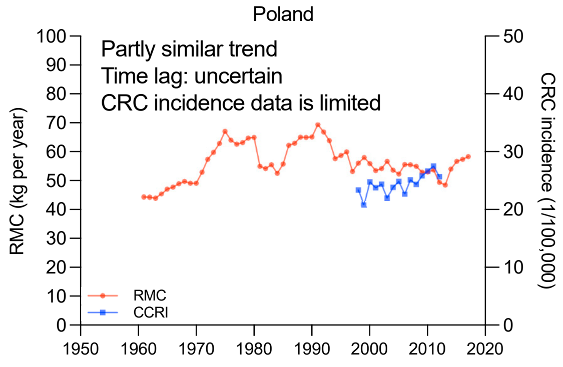

XIV

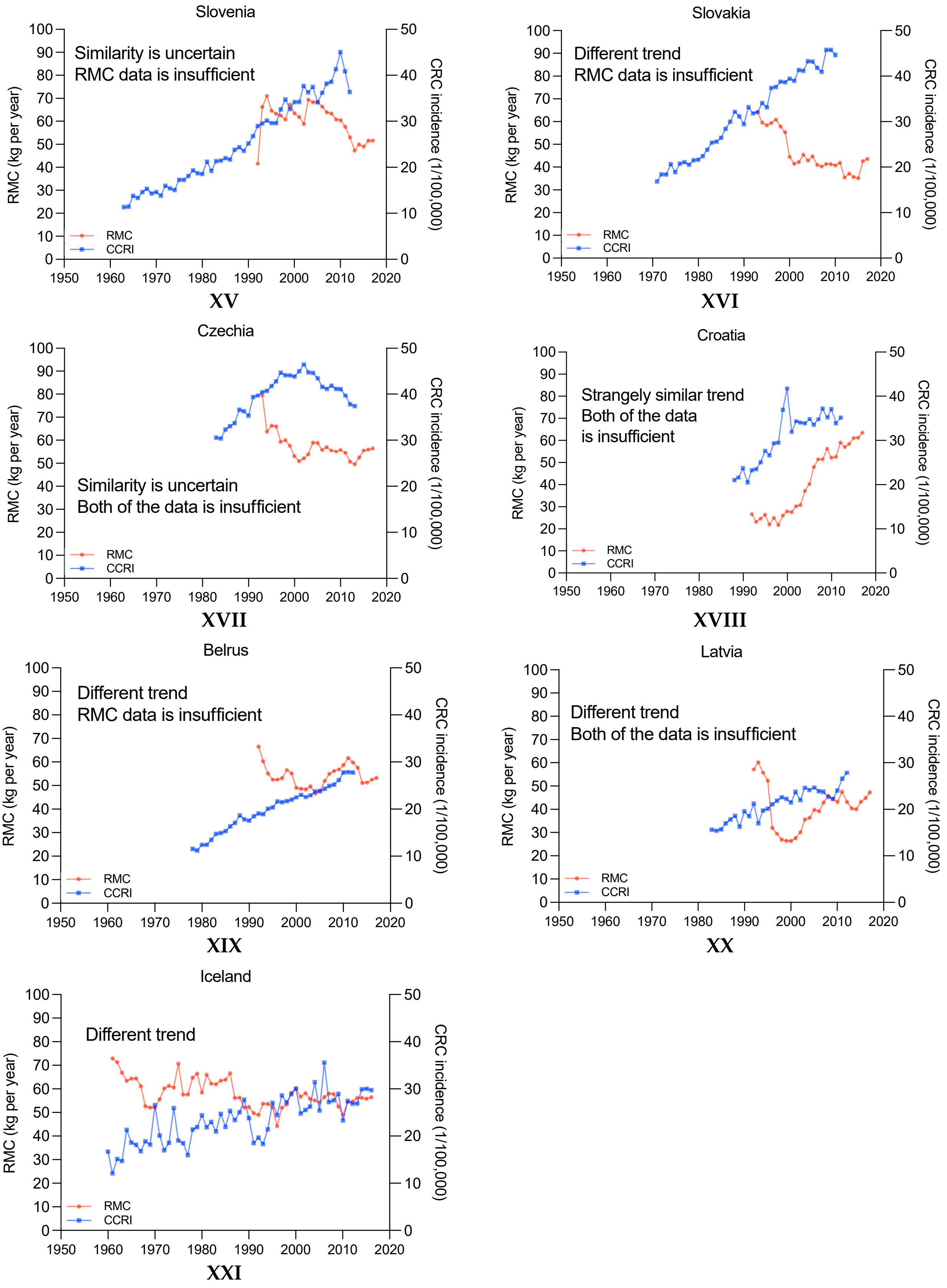

Figure S4. Time Association between RMC changes and CRC incidence changes in countries and regions with maximum annual per capita RMC higher than 60 kg per year.

Table S1. Correlation coefficients between RMC and OCI.

Type	Correlation Coefficient	P-value
Group 1 (n=31)	0.788	0.000
Group 2 (n=32)	0.580	0.001
Group 3 (n=32)	0.752	0.000
Group 4 (n=32)	0.658	0.000
Group 5 (n=32)	0.861	0.000

Data from 159 countries and regions were categorized into five groups based on population size ordering. Correlation coefficients between RMC and OCI were calculated separately. The results based on these five groups of data are consistent with the calculation for the entire sample (0.798, p<0.001, in Section 3.1.1).

Table S2. ANOVA of the cluster.

Type	Cluster		Error		F	P-vaule
	MS	df	MS	df		
Bladder	1159.829	1	11.615	151	99.858	p=0.000
Brain,CNS	182.641	1	3.274	151	55.791	p=0.001
Breast	46110.98	1	171.646	151	268.64	p=0.002
Cervix uteri	2653.608	1	196	151	13.539	p=0.003
Corpus uteri	3200.626	1	20.372	151	157.108	p=0.004
Gallbaldder	1.464	1	1.017	151	1.441	p=0.232
Hodgkin lymphoma	34.772	1	0.514	151	67.625	p=0.000
Hypopharynx	5.105	1	0.507	151	10.078	p=0.002
Kaposi sarcoma	14.262	1	7.533	151	1.893	p=0.171
Kidney	1068.244	1	6.402	151	166.854	p=0.000
Larynx	25.39	1	1.393	151	18.233	p=0.001
Leukaemia	304.324	1	3.746	151	81.246	p=0.002
Lip, oral cavity	61.889	1	3.121	151	19.832	p=0.003
Liver	472.725	1	63.375	151	7.459	p=0.007
Lung	7965.271	1	75.724	151	105.188	p=0.000
Melanoma skin	2950.853	1	28.022	151	105.305	p=0.001
Mesothelioma	5.378	1	0.071	151	75.782	p=0.002
Mutiple myeloma	143.636	1	0.727	151	197.52	p=0.003
Nasopharynx	9.304	1	1.523	151	6.109	p=0.015
Non-hodgkin lymphoma	622.92	1	4.654	151	133.837	p=0.000
Non-melanoma of skin	8319.001	1	254.946	151	32.63	p=0.001
Oesophagus	5.525	1	10.61	151	0.521	p=0.472
Oropharynx	45.998	1	0.64	151	71.888	p=0.000
Ovary	277.295	1	6.079	151	45.612	p=0.001
Pancreas	561.81	1	3.733	151	150.485	p=0.002
Penis	0.959	1	1.039	151	0.924	p=0.338
Prostate	65725.336	1	323.163	151	203.382	p=0.000
Salivary glands	0.01	1	0.091	151	0.115	p=0.735
Stomach	8.674	1	29.622	151	0.293	p=0.589
Testis	618.343	1	4.922	151	125.621	p=0.000
Thyroid	616.202	1	15.401	151	40.011	p=0.000
Vagina	0.008	1	0.067	151	0.123	p=0.727
Vulva	12.917	1	0.451	151	28.615	p=0.000
Colon	4274.019	1	20.065	151	213.007	p=0.001
Rectum	1081.154	1	9.814	151	110.167	p=0.002
Anus	2.108	1	0.17	151	12.405	p=0.001

Table S3. GDP per capita, OCI, RMC, PMC, SFC in 159 countries and regions.

Country name	ISO3	RMC (kg per year)	PMC (kg per year)	SFC (kg per year)	OCI (1/100,000)	GDP per capita (US $)
Norway	NOR	48.68	13.30	50.83	587.52	63400.99
Kuwait	KWT	27.39	52.25	12.69	100.45	52182.33
Switzerland	CHE	56.72	13.98	16.26	402.52	48385.96
United States of America	USA	70.77	49.64	22.37	1221.51	46827.12
Ireland	IRL	62.03	26.58	21.06	430.41	41997.98
Australia	AUS	75.07	37.40	24.31	945.43	40018.90
Denmark	DNK	62.35	19.42	23.40	438.24	39982.15
Netherlands	NLD	60.06	18.38	20.28	437.71	39166.50
Canada	CAN	59.64	35.24	23.43	365.55	38243.56
Sweden	SWE	58.34	12.83	30.59	382.05	36893.81
Germany	DEU	68.56	15.13	14.16	354.93	36474.24
Austria	AUT	76.62	17.21	12.34	316.46	36453.57
Iceland	ISL	54.64	18.35	91.45	433.72	34852.42
Japan	JPN	28.22	16.14	59.50	275.94	34202.50
France	FRA	64.26	23.94	33.15	390.28	34012.14
Finland	FIN	52.82	15.48	33.59	390.65	33659.75
Saudi Arabia	SAU	10.78	37.43	8.85	114.91	33268.51
United Kingdom of Great Britain and Northern Ireland	GBR	48.93	28.57	20.11	486.56	33105.29
Italy	ITA	62.69	17.93	24.96	381.81	32559.55
Taiwan	TWN	46.16	32.41	32.90	274.60	30874.74
Oman	OMN	21.39	22.54	26.51	111.72	29910.23
New Zealand	NZL	70.95	27.66	24.95	860.11	28864.18
Spain	ESP	73.19	27.21	42.18	356.70	28013.10
Republic of Korea	KOR	38.08	12.73	53.40	246.45	26676.98
Israel	ISR	27.28	61.26	22.91	328.08	26526.05
Trinidad and Tobago	TTO	12.67	41.44	17.53	186.85	23788.78
Cyprus	CYP	53.01	27.14	20.28	278.39	23506.69
Portugal	PRT	58.70	26.10	56.60	284.55	23212.44
Slovenia	SVN	59.68	22.85	8.88	339.62	23027.30
Greece	GRC	60.51	15.08	20.84	354.73	22413.25
Malta	MLT	55.82	19.82	29.94	310.46	21695.92
Estonia	EST	43.84	16.49	16.05	311.77	19582.98
Chile	CHL	39.93	29.27	14.85	219.33	17118.24
Hungary	HUN	54.90	27.40	4.62	384.34	16540.48
Poland	POL	55.68	19.79	10.16	271.96	16150.36
Russian Federation	RUS	38.43	18.34	19.86	293.52	16089.98
Argentina	ARG	65.11	29.58	7.99	234.01	16031.95
Malaysia	MYS	15.61	35.86	57.64	162.02	16025.05
Croatia	HRV	40.65	8.64	12.29	355.76	15910.46
Lithuania	LTU	47.94	17.70	32.11	301.21	15752.00
Venezuela	VEN	25.54	30.29	15.22	263.11	15655.24
Latvia	LVA	40.37	15.15	20.73	277.06	15264.69
Kazakhstan	KAZ	41.00	9.88	3.44	245.52	15171.70
Mauritius	MUS	13.69	27.77	20.79	145.31	15116.47
Uruguay	URY	58.29	13.99	6.95	299.67	14529.08
Turkey	TUR	12.51	13.54	7.39	205.82	14147.34
Mexico	MEX	31.13	25.39	11.83	322.31	13334.61
Panama	PAN	29.71	26.69	13.01	176.96	13046.97
Gabon	GAB	18.35	20.96	38.42	176.19	13039.46
Belarus	BLR	54.10	16.70	11.87	253.56	12835.90
Iran	IRN	11.47	18.20	7.09	153.00	12455.52
Barbados	BRB	28.07	43.05	36.54	275.99	12049.83
Bulgaria	BGR	36.64	17.37	4.45	274.15	11765.71
Thailand	THA	15.05	13.01	28.99	175.46	11763.27
Romania	ROU	40.44	17.10	4.53	266.68	11670.70
Brazil	BRA	47.94	32.22	7.17	275.31	11604.19
Costa Rica	CRI	24.70	20.67	8.75	248.28	10704.75
Turkmenistan	TKM	39.96	3.03	3.32	177.44	10555.73

Botswana	BWA	14.66	4.74	3.64	179.79	10221.48
Colombia	COL	22.11	20.23	5.53	233.31	10147.41
Lebanon	LBN	18.50	20.27	7.36	267.71	10102.51
Azerbaijan	AZE	15.27	6.84	2.02	213.38	9482.02
Algeria	DZA	11.51	7.62	4.21	102.54	9462.39
North Macedonia	MKD	21.21	14.25	4.98	253.42	9370.18
Jordan	JOR	10.38	24.71	4.86	132.81	9321.17
South Africa	ZAF	22.93	25.54	7.25	265.25	9255.46
Dominican Republic	DOM	18.29	26.41	8.79	149.19	8745.37
Tunisia	TUN	11.87	12.52	11.37	133.44	8732.69
Ecuador	ECU	27.11	16.77	7.44	184.57	8448.65
Saint Lucia	LCA	30.87	60.43	28.94	215.86	8287.20
Egypt	EGY	11.77	9.45	15.93	106.80	8089.76
Peru	PER	8.04	16.72	21.29	169.54	7811.18
Ukraine	UKR	28.34	14.31	12.58	331.43	7784.02
Bosnia and Herzegovina	BIH	15.70	9.49	3.94	247.09	7380.35
Armenia	ARM	22.20	8.82	2.35	244.96	7241.49
China	CHN	36.50	9.84	27.17	206.52	7231.85
Indonesia	IDN	4.92	5.44	24.87	135.40	7144.07
Sri Lanka	LKA	1.78	4.77	23.16	115.32	7107.28
Namibia	NAM	16.54	9.41	13.43	160.93	7037.60
Iraq	IRQ	3.90	6.00	2.20	109.61	6931.75
Georgia	GEO	19.86	8.79	5.37	217.95	6920.67
Dominica	DMA	24.45	37.71	29.99	251.32	6915.86
Jamaica	JAM	11.28	44.34	23.27	202.74	6857.41
Albania	ALB	28.72	9.38	4.60	205.69	6721.11
Paraguay	PRY	47.01	7.06	4.55	150.30	6359.63
El Salvador	SLV	9.80	14.66	5.32	205.14	6276.23
Uzbekistan	UZB	26.46	1.54	0.83	168.66	6240.95
Guatemala	GTM	11.33	14.40	1.98	197.48	6202.75
Eswatini	SWZ	23.67	5.82	4.02	240.83	5951.78
Mongolia	MNG	69.64	0.90	0.38	334.19	5940.64
Morocco	MAR	10.50	13.36	11.84	110.00	5910.76
Cuba	CUB	20.96	14.17	9.04	258.95	5172.08
Philippines	PHL	21.16	8.83	31.00	142.43	4963.45
Congo	COG	6.15	8.64	23.10	192.23	4853.65
Bolivia	BOL	31.99	24.44	1.83	233.20	4722.29
Cabo Verde	CPV	18.49	10.37	15.49	145.49	4530.33
Angola	AGO	11.71	6.51	14.24	170.89	4509.14
Republic of Moldova	MDA	18.29	10.46	7.64	218.63	4349.96
Yemen	YEM	6.94	8.41	5.31	114.77	3996.93
Honduras	HND	11.36	15.41	3.23	176.47	3859.95
Pakistan	PAK	10.45	3.26	2.07	174.08	3816.71
Kyrgyzstan	KGZ	31.71	3.57	1.36	180.76	3772.91
Vietnam	VNM	28.74	6.75	25.10	168.60	3707.98
India	IND	2.64	1.40	5.07	99.61	3696.26
Nigeria	NGA	6.14	1.49	10.47	137.70	3448.32
Nicaragua	NIC	6.71	13.12	3.81	176.49	3415.51
Laos	LAO	14.44	3.20	15.92	149.79	3251.22
Myanmar	MMR	11.69	13.29	29.78	191.14	2929.17
Sao Tome and Principe	STP	4.73	6.63	27.92	145.90	2699.62
Ghana	GHA	3.57	3.94	26.63	147.84	2619.56
Tajikistan	TJK	10.93	1.65	0.29	155.11	2569.40
Cote d”Ivoire	CIV	3.80	1.70	15.10	120.31	2476.31
Mauritania	MRT	18.66	3.27	8.87	146.75	2473.92
Djibouti	DJI	14.34	2.35	2.78	170.57	2339.86
Kenya	KEN	12.28	0.71	4.18	134.86	2273.54
Cameroon	CMR	8.27	2.87	15.14	163.42	2194.49
Zambia	ZMB	9.09	3.17	7.97	180.51	2179.31
Lesotho	LSO	11.62	4.60	0.85	216.87	2165.42
Cambodia	KHM	12.98	2.00	26.84	147.91	2126.28
Bangladesh	BGD	2.48	1.21	15.83	101.52	2083.02

Senegal	SEN	8.78	3.48	26.84	147.75	2074.98
Benin	BEN	4.83	10.69	11.87	142.06	1900.94
Nepal	NPL	9.89	0.86	1.72	93.78	1890.24
Zimbabwe	ZWE	10.39	3.86	2.45	212.85	1787.15
North Korea	PRK	7.19	1.62	11.15	172.09	1767.51
United Republic of Tanzania	TZA	7.69	1.59	8.07	143.06	1625.49
Gambia	GMB	4.00	2.98	23.99	129.93	1548.21
Haiti	HTI	8.86	4.46	3.60	206.17	1542.32
Uganda	UGA	9.48	1.76	11.00	173.64	1466.95
Mali	MLI	14.51	2.72	8.37	149.07	1347.77
Burkina Faso	BFA	12.04	2.30	3.68	161.43	1320.04
Sierra Leone	SLE	2.30	2.54	21.03	144.03	1319.88
Guinea-Bissau	GNB	14.55	1.56	2.79	175.81	1296.81
Madagascar	MDG	11.66	3.61	6.41	143.55	1267.22
Rwanda	RWA	4.26	0.59	2.43	161.70	1263.70
Chad	TCD	20.61	0.52	6.63	140.29	1261.73
Togo	TGO	4.46	4.59	10.41	135.79	1196.39
Guinea	GIN	6.38	1.14	10.94	171.71	1166.57
Mozambique	MOZ	6.71	2.25	5.21	164.50	1160.48
Afghanistan	AFG	11.67	1.34	0.11	176.45	1132.99
Malawi	MWI	6.07	2.33	6.29	161.40	985.69
Central African Republic	CAF	25.35	1.24	6.49	184.32	851.43
Liberia	LBR	4.10	5.50	5.21	135.00	799.50
Niger	NER	12.98	0.98	1.72	140.37	788.12
United Arab Emirates	ARE	22.62	36.55	24.12	204.82	N/A
Antigua and Barbuda	ATG	19.58	55.70	49.23	223.99	N/A
Bahamas	BHS	49.70	48.91	29.89	262.19	N/A
Belize	BLZ	17.68	25.59	13.47	162.14	N/A
Fiji	FJI	24.64	17.01	33.31	163.80	N/A
Grenada	GRD	18.88	32.75	30.04	216.81	N/A
Guyana	GUY	6.11	29.65	37.80	157.49	N/A
Kiribati	KIR	14.48	14.36	74.01	188.38	N/A
Maldives	MDV	5.28	8.76	148.69	109.40	N/A
Solomon Islands	SLB	8.40	2.01	36.57	162.99	N/A
Suriname	SUR	12.23	28.85	15.43	165.34	N/A
Timor-Leste	TLS	12.23	5.20	3.59	120.18	N/A
Saint Vincent and the Grenadines	VCT	20.76	58.85	16.87	220.68	N/A
Vanuatu	VUT	25.90	8.19	32.02	217.23	N/A
Samoa	WSM	43.28	38.16	48.90	157.07	N/A
